# Effects of COVID-19 e-mental health interventions: A systematic review of systematic reviews and meta-analyses

**DOI:** 10.1016/j.invent.2025.100802

**Published:** 2025-01-18

**Authors:** Romy RW, Xiaoli Nan

**Affiliations:** a Loyola Marymount University, United States of America; b University of Maryland, College Park, United States of America

**Keywords:** E-mental health interventions, COVID-19, Systematic review, Videoconferencing, Anxiety

## Abstract

The COVID-19 pandemic has had a profound impact on global mental health. E-mental health has the potential to enhance the quality of care and can be swiftly implemented on a large scale. We performed a systematic review of systematic reviews, including meta-analyses, to assess the effects of COVID-19 e-mental health interventions. We followed an established search, screening, coding, and reporting protocol. Methodological quality was evaluated using the Measurement Tool to Assess Systematic Reviews (AMSTAR-2) checklist. The searches resulted in a total of 2341 articles. Of these, twelve systematic and meta-analytic reviews were included. The findings indicated that cognitive behavioral therapy (CBT) and psychoeducation were the most used mental health intervention types. E-mental health interventions were delivered via various communication channels including videoconferencing, telephone-based approaches, and mobile applications. E-mental health interventions have demonstrated their effectiveness in addressing prevalent mental health issues, particularly anxiety, depression, and stress. This study underscores the importance of e-mental health interventions in enhancing accessibility and efficiency to reduce mental health symptoms, providing valuable insights for policymakers and clinicians addressing mental health challenges exacerbated by global pandemics.

## Highlights

• Cognitive behavioral therapy (CBT) and psychoeducation were the most used mental health intervention types.
• E-mental health interventions were delivered via various communication channels including videoconferencing, telephone-based approaches, and online applications (e.g., smartphone apps and virtual support groups).
• E-mental health interventions have demonstrated their effectiveness in addressing prevalent mental health issues, particularly anxiety, depression, and stress.
• The importance of e-mental health interventions in enhancing accessibility and efficiency to reduce mental health symptoms.

## 1 Introduction

Advancements in technology are reshaping the landscape of communication. E-mental health has emerged as a crucial service embraced by numerous counselors and their clients, as noted by the American Psychiatric Association (2017). It entails “a mental health intervention between a patient (or a group of patients) and a therapist, utilizing technology as the mode of communication” (Barak & Grohol, 2011, p. 157), such as email, synchronous chat, phone, videoconferencing (Mallen et al., 2003). The COVID-19 pandemic has had a profound impact on global mental health, leading to heightened levels of stress, anxiety, depression, and loneliness (World Health Organization, 2022a). In the post-pandemic landscape, it is crucial to prioritize the mental well-being of individuals and communities. Additionally, the COVID-19 pandemic has radically altered how counselors are using technology to provide safe spaces, intimate connections, and relational continuity. In order to follow guidelines for social distance practicing, many counselors have had to switch traditional face-to-face counseling to full-time telehealth (American Psychiatric Association, 2020). The Centers for Medicare and Medicaid Services (2021) announced COVID-19 Emergency Declaration Blanket Waivers for Health Care Providers in order to increase flexibility for online counseling services. According to the American Psychiatric Association (2021), approximately 38 % of Americans have utilized telehealth services to consult with medical or mental health professionals, with videoconferencing being the preferred mode of delivery. Consequently, the heightened accessibility and adoption of technology in counseling have opened up novel avenues for therapists and clients to connect and interact.

It is widely acknowledged that e-mental health has the potential to enhance the quality of care, particularly during the COVID-19 pandemic. Quality in e-mental health interventions can be maintained in terms of intervention process and outcomes, such as self-disclosure, working alliance, and clients' satisfaction. Research has shown that there was a higher level of self-disclosure in online synchronous chat than face-to-face mental health intervention because it helps with clients' self-expression during the treatment process (Joinson, 2001). Mental health intervention using phone indicated a similar level of clients' satisfaction compared with a face-to-face setting (Sangha et al., 2003). Cook and Doyle (2002) found that participants reported higher working alliance in asynchronous and synchronous text-based counseling than face-to-face treatment. Additionally, e-mental health interventions can be swiftly implemented on a large scale compared with traditional face-to-face mental health interventions. E-mental health interventions offer scalable and accessible treatment options, enabling personalized care that addresses mental health inequities, geographic barriers, socioeconomic status, and stigma (Gratzer et al., 2020). Many healthcare organizations have adopted virtual platforms for greater accessibility and cost-effectiveness, significantly increasing patient visits (Wright et al., 2019).

However, there remains a notable gap in the availability of robust evidence concerning its effectiveness and cost-effectiveness across various domains. As the body of evidence in this domain continues to expand, numerous systematic reviews have already been conducted. A comprehensive assessment of this accumulating evidence is crucial for informing both clinical practice and health policy decisions (Whitlock et al., 2008). Thus, in this context, there arises a need for through reviews that can appraise a multitude of interventions, diverse populations, and a range of outcomes. Therefore, the aim of this study is to undertake a systematic review of systematic reviews, with a focus on evaluating the effectiveness of e-mental health interventions across various intervention modalities and outcomes during the COVID-19 pandemic. This time period is of particular interest for several reasons. The COVID-19 pandemic caused a significant 25 % global increase in mental health issues, driven by unprecedented stressors such as social isolation, fear of infection, bereavement, and financial uncertainty, all contributing to heightened anxiety and depression (World Health Organization, 2022b). Second, the COVID-19 pandemic necessitated a rapid shift in mental health service delivery, with many counselors transitioning from traditional face-to-face counseling to exclusively using e-mental health practices to comply with social distancing guidelines, making e-mental health interventions the primary means of providing care and ensuring access to support during a time of heightened mental health needs (American Psychiatric Association, 2020). Third, research on e-mental health interventions has surged in response to the escalating mental health challenges during the COVID-19 pandemic. The practice of conducting systematic reviews of systematic reviews offers a comprehensive and up-to-date perspective on the rapidly evolving landscape of e-mental health. This methodology not only ensures a high level of evidence by consolidating findings from multiple systematic reviews, thereby bolstering the strength and dependability of the conclusions but also illuminates potential gaps and disparities in the existing body of literature, guiding researchers toward unexplored avenues for further inquiry.

## 2 Methods

### 2.1 Search strategy and inclusion criteria

A literature search was performed on seven databases in EBSCO (APA PsycInfo, Academic Search Ultimate, Communication & Mass Media Complete, Education Source, Health Source: Nursing/Academic Edition, MEDLINE, and Psychology and Behavioral Sciences Collection) and five Web of Science databases (Science Citation Index Expanded, Social Sciences Citation Index, Arts & Humanities Citation Index, Emerging Sources Citation Index, and Book Citation Index) in January 2023. The search was limited to articles published in English between 2020 and 2023. The following search string was used: (“mental health” AND (covid-19 OR coronavirus OR sars-cov-2) AND (“scoping review” OR “systematic review” OR “meta-analysis” OR “meta-analytic”). This study was guided by the preferred reporting items for systematic review and meta-analysis (PRISMA) statement (Page et al., 2021).

### 2.2 Study selection

The systematic reviews and meta-analyses included in this analysis focused on assessing the effectiveness of e-mental health interventions. The inclusion criteria for these reviews were as follows: (1) reviews related to mental health interventions, (2) reviews conducted during the COVID-19 pandemic, (3) reviews categorized as systematic reviews or meta-analyses, and (4) reviews published after the year 2019.

The initial screening process was based on the titles and abstracts of the articles. During this stage, articles were independently assessed by the two authors to determine their potential relevance. Abstracts that lacked sufficient information for evaluation were retrieved for a comprehensive full-text review. Subsequently, the two authors independently evaluated the full-text articles to determine their eligibility for inclusion in the analysis.

### 2.3 Data extraction and quality assessment

Study information including year of publication and publication type (e.g., journal articles and theses) were automatically generated by Zotero. The two authors coded other study information including: author, year, title, journal, publication date, review type, literature type, language of the included articles, organizing theory/model, initial records retrieved, number of publications included, number of studies/comparisons included, number of studies/comparisons included, type of population, countries/regions, design of studies, intervention type, units of analysis (meta-analysis), intervention effects analyzed (meta-analysis only)-the comparison group, effect size parameter (meta-analysis only), outcome measures, outcome measures time frame, results, moderators, and conclusions. To achieve consensus among coders, five reviews were randomly selected for coder training. Two authors independently coded the five reviews and then met to discuss any discrepancies. After reaching agreement, the two authors independently coded the 12 reviews. The coding achieved a high intercoder reliability, with a Krippendorff's alpha ranging from 0.82 to 1.00. Reviews were evaluated using the Measurement Tool to Assess Systematic Reviews (AMSTAR-2, Shea et al., 2017) checklist for assessing methodological quality.

## 3 Results

**Fig. 1:**
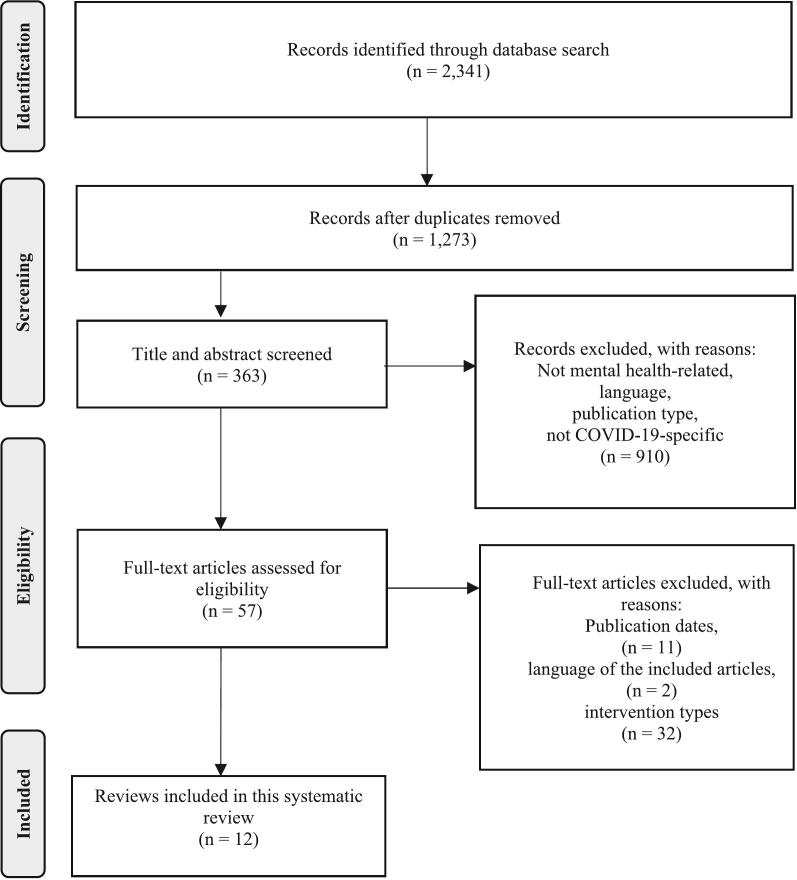
Flow of information through the different phases of the systematic review. For more information, visit: http://www.prisma-statement.org/.

A flow diagram of literature search and study selection results is shown in Fig. 1. The database search resulted in 2341 articles. After exclusion of duplicates, there were 1273 articles. A total of 910 articles were excluded in title and abstract screening for several reasons. Records not focused on mental health were removed to maintain relevance to the study's core topic. Non-English studies were excluded due to language constraints. Certain publication types, such as book chapters and conference abstracts, were also excluded. Additionally, records that did not specifically address COVID-19 (e.g., studies on H1N1) were excluded to remain the focus to the COVID-19 pandemic. Full text of 57 eligible articles were reviewed. Out of these, 45 were excluded for not meeting the criteria relating to publication date, language of the included reviews, or intervention types. The final dataset for this systematic review included 12 articles.

### 3.1 General characteristics of reviewed papers

There are a total of twelve articles comprising six systematic reviews and six meta-analyses (see the Supplementary material). Among these, two articles explicitly stated that the reviews they included underwent a peer review process, while three articles included a mix of peer-reviewed and non-peer-reviewed reviews. The remaining seven articles did not provide information regarding the peer-review status of the included reviews. Regarding the language of the included reviews, eight articles reported that they exclusively included English-language reviews. Three articles included a combination of both English and non-English articles, and one article did not specify the language criteria for their included reviews. Two of the articles framed their reviews using theoretical models: one employed the National Quality Framework (NQF, Abraham et al., 2021), while the other utilized the E-health typology (Ellis et al., 2021). In terms of the initial number of records retrieved during the search, this ranged from 291 to 45,777 articles. Furthermore, two of the articles explicitly specified the target population of their reviews, one focusing on university students (Malinauskas and Malinauskiene, 2022), and the other concentrating on COVID-19 patients and medical staff (D'Onofrio et al., 2022). Lastly, in terms of research methods, eight of the articles employed a quantitative approach, three adopted a qualitative methodology, and one article utilized a mixed-methods approach.

### 3.2 Meta-analysis reviews

Four reviews conducted comparisons between intervention conditions and control conditions (Komariah et al., 2022; Kurniawan et al., 2022; Witarto et al., 2022; Ye et al., 2022). Additionally, one article compared an intervention with no treatment/usual care (Malinauskas and Malinauskiene, 2022), while another article employed a pretest to posttest design (D'Onofrio et al., 2022). In terms of research design, six articles included only randomized controlled trials (RCT; Bonardi et al., 2022; Komariah et al., 2022; Kurniawan et al., 2022; Malinauskas and Malinauskiene, 2022; Witarto et al., 2022), while five articles included a combination of RCTs and non-RCTs (Ali et al., 2021; Abraham et al., 2021; D'Onofrio et al., 2022; Ellis et al., 2021; Hatami et al., 2022; Siegel et al., 2021). Notably, one article did not provide information about the specific research design of included articles. Six articles reported effect size d and others did not report effect size. Three articles tested moderators including control group, region, and duration of intervention (Witarto et al., 2022), clinically guided inference-based CBT versus self-guided inference-based CBT (Komariah et al., 2022), and types of CBT (Ye et al., 2022).

### 3.3 Critical appraisal of included reviews

We performed a rigorous evaluation of the included reviews using the AMSTAR-2 criteria, consisting of 16 items (Shea et al., 2017). This evaluation aims to present the outcomes of our quality assessment of the 12 systematic reviews (Appendix A). The articles ranged from critically poor to moderate quality, indicating the presence of significant methodological deficiencies across all 12 currently published reviews that evaluate the effectiveness of e-mental health interventions.

Table 1 provides an overview of the main findings, followed by more detailed result summary in the main text.

**Table 1:** The main findings.

Aspects	Key findings
Intervention types	CBT identified as the primary e-mental health intervention. CBT consistently effective in reducing mental health symptoms during the COVID-19 pandemic. Psychoeducation, particularly online mindfulness interventions, gained prominence with mixed findings on their efficacy. Crisis intervention and online support group interventions have also shown their efficacy in alleviating anxiety and depression.
Channel modality	Various communication channels used for e-mental health interventions. Videoconferencing was the most frequently utilized communication channel. Telephone was the second most common method. Mobile applications were used extensively. Exploration of alternative channels (e.g., virtual reality) required for a deeper understanding of their efficacy.
Outcome measures	Emphasis on anxiety, depression, and stress as primary mental health outcomes. These outcomes critical during the COVID-19 pandemic. E-mental health interventions consistently effective in addressing these prevalent mental health challenges.

### 3.4 Intervention types

#### 3.4.1 Cognitive behavioral therapy

In the realm of mental health interventions, a diverse array of strategies is employed to address the multifaceted spectrum of mental health concerns. Among these approaches, cognitive behavioral therapy (CBT), a well-established form of psychotherapy, emerged as the predominant e-mental health intervention across all reviews. Its effectiveness in alleviating mental health symptoms was consistently observed. Bonardi et al. (2022) highlighted a significant decrease in mental health symptoms with remote psychotherapy during the initial weeks of the COVID-19 lockdown compared to the pre-pandemic months. Moreover, Komariah et al. (2022) conducted a comprehensive analysis of pre- and post-online CBT interventions for depression using Patient Health Questionnaire-9 (PHQ-9) scale, revealing a significant effect (*p* < .001) with a pooled mean difference (MD) of 3.90, 95 % CI: 3.60–4.62), *I*^2^ = 93 %. Similarly, for anxiety using the General Anxiety Disorder-7 (GAD-7) scale, the pre- and post-CBT intervention showed a significant effect (*p* < .001) with a pooled MD of 5.67, 95 % CI: 4.49–6.85, *I*^2^ = 95 %.

However, it is worth noting that substantial heterogeneity was also observed in the pre-/post-depression and anxiety forest plots (I^2^ = 93 %; *p* < .001 and I^2^ = 90 %, respectively), indicating variations in the study outcomes. Ye et al. (2022) provided further support for the effectiveness of online CBT, particularly in reducing anxiety, with a significant standardized mean difference (SMD) of −0.86 and 95 % CI [−1.49, −0.22], I^2^ = 95.67 % and 95 % CI [92.41, 97.53], *p* < .001). These findings collectively emphasize the pivotal role of CBT as a powerful e-mental health intervention in ameliorating mental health symptoms during the COVID-19 pandemic.

#### 3.4.2 Psychoeducation

The second commonly employed type is psychoeducation among all the reviews. Psychoeducation is a process aimed at educating individuals with mental health concerns and their families about the intricacies of their conditions, encompassing etiology, progression, consequences, prognosis, treatment options, and alternatives (Barker, 2003). This approach includes mindfulness-related programs, incorporating guided meditation exercises, educational videos, and discussion forums to actively engage participants (Zenner et al., 2014).

Bonardi et al. (2022) examined the impact of psychoeducation in a lay-delivered telephone intervention and a peer-moderated education and support intervention. Both interventions demonstrated significant improvements in anxiety (SMD 0.35, 95 % CI [0.09 to 0.60]; SMD = 0.31, 95 % CI [0.03 to 0.58]) and depressive symptoms (SMD 0.31, 95 % CI [0.05 to 0.56]; SMD = 0.31, 95 % CI [0.07 to 0.55] at the 6-week post-intervention assessment. However, Ye et al. (2022) provided a contrasting perspective, reporting that psychoeducation did not demonstrate significant effectiveness (SMD = −0.25 and 95 % CI [−0.39, −0.11], *I*^2^ = 0.00 %, 95 % CI [0.00, 89.60], *p* = .642).

Other e-mental health intervention types have demonstrated their efficacy in alleviating mental health outcomes. For example, in Ali et al. (2021) systematic review, one study indicated that after 1 week hospitalization with comprehensive crisis intervention, the scores of anxiety and depression were significantly decreased among the patients (Yang et al., 2020). Additionally, in Bonardi et al.'s (2022) systematic review, online videoconferencing support group interventions (Bonardi et al., 2022) indicated efficacy in loneliness (4 weeks: SMD = 0.09, 95 % CI [0.12, 0.31], 10 weeks SMD = 0.02 95 % CI [−0.22, 0.26]). These e-interventions further contribute to the diverse landscape of e-mental health interventions.

### 3.5 Communication modality

We examine the various communication modalities of e-mental health intervention in the context of e-mental health interventions during the COVID-19 pandemic.

#### 3.5.1 Videoconferencing

Videoconferencing has emerged as the predominant modality for delivering e-mental health interventions. Ellis et al. (2021) demonstrated that videoconferencing psychotherapy is equally effective as face-to-face intervention for individuals with panic disorder and agoraphobia, highlighting its efficacy. Hatami et al.'s (2022) systematic review found that all seven included reviews utilized videoconferencing interventions, all of which yielded significant positive effects. Additionally, Witarto et al.'s (2022) review showed that videoconferencing reduced anxiety (g = −0.46, 95 % CI [−0.74, −0.19], Z = 3.34, *p* < .001), depression (g = −0.35, 95 % CI [−0.62, −0.08], Z = 2.52, *p* = .01), and stress (g = −0.81, 95 % CI [−1.64, 0.01], Z = 1.93, *p* = .05).

#### 3.5.2 Telephone

The use of telephone-based interventions has emerged as the second most prevalent choice following videoconferencing. Ali et al. (2021) reported a significant surge in telephone utilization (*p* < .001) during the initial weeks of the COVID-19 lockdown compared to the period prior to the pandemic. Additionally, Bonardi et al. (2022) identified that telephone interventions resulted in a notable reduction in anxiety and depression symptoms and a marked improvement in mental health. The effect sizes varied from SMD = 0.31, 95 % CI [0.05 to 0.56] to SMD = 0.46, 95 % CI [0.20 to 0.72] among participants. As highlighted by Siegel et al. (2021) in their review study, families hesitant to adopt video conferencing often express a preference for telephone-based care, particularly when privacy concerns, technological limitations, or financial constraints impede the use of video conferencing platforms. It's worth noting that patients with lower socioeconomic status tend to favor telephone care. Nevertheless, maintaining a therapeutic alliance exclusively over the phone can be challenging due to its impersonal nature and the absence of visual cues. To address this, Siegel et al. (2021) recommend comprehensive training for clinicians, including adaptations for the effective delivery of telehealth services, especially in the context of telephone-based care.

#### 3.5.3 Mobile applications

Ellis et al. (2021) summarized that their included reviews utilized smartphone apps and virtual support groups. They found that self-directed treatment apps may be particularly beneficial for individuals who prefer seeking assistance anonymously and value flexibility, as well as for those who have historically encountered challenges in engaging with traditional psychological services. According to Hatami et al. (2022), online guided group sessions through a mobile application have shown significant reductions in loneliness (*p* = .02) and depressive symptoms (*p* = .05). Their findings revealed that the “Gro health app” resulted in a substantial decrease in depression (*p* < .001), anxiety (*p* < .001), and stress (*p* < .001) among participants following the intervention. They also noted that the only study that did not produce significant effects utilized an asynchronous intervention (Psycovid app). Witarto et al.'s (2022) review demonstrated that online mindfulness applications effectively reduced depression (g = −0.29, 95 % CI [−0.53, −0.05], Z = 2.40, *p* = .002), though the same effectiveness was not observed for anxiety and stress reduction.

Wearable devices, virtual reality, artificial intelligence, and therapeutic gaming represent emerging digital health interventions with significant potential to improve mental health (Ellis et al., 2021). However, none of the studies in our final dataset specifically examined these modalities, the current evidence supporting their effectiveness remains limited. Despite this gap, these technologies are expected to become increasingly essential in digital health, as the field continues to evolve (Ellis et al., 2021).

### 3.6 Outcome measures

#### 3.6.1 Anxiety

All 12 articles focused on using e-mental health to treat anxiety. Bonardi et al. (2022) discovered that three reported interventions (30–32), designed specifically for COVID-19, demonstrated a reduction in general or COVID-19-specific anxiety symptoms compared to no intervention or waitlist control. The effect sizes ranged from SMD = 0.31, 95 % CI [0.03 to 0.58] to 0.74, 95 % CI [0.58 to 0.90] at the last trial assessments. In contrast, for the six trials with a high risk of bias and reporting concerns (33–38), the reported effects on anxiety and depression symptoms ranged from SMD = 0.78, 95 % CI [0.17 to 1.38] to 1.14, 95 % CI [0.80 to 1.49] for individual and group CBT when compared to control group.

D'Onofrio et al.'s (2022) meta-analysis indicated significant reductions in anxiety, depression, functional impairment, intolerance of uncertainty, and insomnia, as well as an increase in coping skills following e-mental health interventions (effect sizes ranged from *d* = 0.21 to 2.89). One of the reviews included in the analysis reported that anxiety significantly decreased (*F* = 26.58, *p* < .001), with a main effect of the group (*F* = 5.634, *p* = .026), and a group-by-time interaction (*F* = 3.743, *p* = .031) in post hoc analyses of individual time points.

Hatami et al.'s (2022) systematic review summarized that one study demonstrated a significant reduction in general anxiety disorder among at-risk older adults after receiving telephone mental health intervention for four weeks (*p* < .001). Another study in Hatami et al.'s review reported a greater reduction in anxiety and depression in the intervention group (*p* = .049) compared to the control group (*p* = .02). In another study, participants used the Gro health app for 12 weeks, resulting in significant reductions in anxiety, perceived stress, and depression (*p* < .001).

Komariah et al.'s (2022) meta-analysis assessed the effectiveness of e-mental health interventions in reducing anxiety and reported a significant decrease between pre- and post-intervention (*p* < .001) with a pooled mean difference (MD) of 5.67, 95 % CI [4.49–6.85], *I*^2^ = 95 %.

Kurniawan et al.'s (2022) meta-analysis specifically examined the impact of e-mental health interventions on reducing anxiety. Their study found that online-based interventions led to a significant reduction in general anxiety disorder scores (pooled MD of 1.30; 95 % CI [2.83–4.65]; *p* < .001) when comparing pre- and post-test scores in the intervention group. Additionally, the intervention group performed significantly better than the control group (pooled MD: −7.26; 95 % CI [−11.58, −2.95]; *p* = .001).

Malinauskas and Malinauskiene's (2022) review reported that nine randomized controlled trials (RCTs) showed no significant difference in anxiety outcomes between the e-mental health intervention treatment group and the control group at post-test (SMD: −0.65; 95 % CI [−1.32, 0.02]). However, there was high heterogeneity in the estimates of anxiety indicators and effect sizes across reviews, *I*^2^ = 97.86 %, *p* < .001; Q = 131.78).

Witarto et al.'s (2022) meta-analysis focused on the effectiveness of online mindfulness-based interventions on mental health. Their findings revealed a statistically significant small effect on anxiety (g = −0.25; 95 % CI [−0.43, −0.06]; *I*^2^ = 27 %). Additionally, they observed significant small effects at follow-up for anxiety (g = −0.28; 95 % CI [−0.48, −0.08]; *I*^2^ = 0 %).

Ye et al.'s (2022) meta-analysis demonstrated that psychosocial interventions were effective in reducing anxiety symptoms compared to controls (SMD = −0.78; 95 % CI [−1.13, −0.44]; *I*^2^ = 94.99 %; 95 % CI [93.25, 96.28]; *p* < .001).

#### 3.6.2 Depression

Depression was the second most frequently addressed mental health outcome in the reviews. Bonardi et al. (2022) discovered that symptoms of depression were reduced, with effect sizes ranging from SMD = 0.31 (95 % CI, 0.05 to 0.56) to 0.38 (95 % CI, 0.22 to 0.55). For the six trials with a high risk of bias and reporting concerns (33–38), the reported effects on symptoms of anxiety and depression ranged from SMD = 0.78 (95 % CI, 0.17 to 1.38) to 1.14 (95 % CI, 0.80 to 1.49) for individual and group cognitive-behavioral therapy interventions when compared to minimal or no intervention.

In D'Onofrio et al.'s (2022) meta-analysis, one of the included reviews reported that post hoc analyses of individual time points showed a significant reduction in depression (*F* = 37.35, *p* < .001), with a main effect of the group (*F* = 4.384, *p* = .047), and a group-by-time interaction (*F* = 5.268, *p* = .009).

Hatami et al.'s (2022) systematic review summarized that one study found a significant reduction in depression (*p* < .001) among at-risk older adults after receiving telephone mental health intervention for four weeks. Another study included in Hatami et al.'s review demonstrated a significant reduction in depressive symptoms (*p* = .05) among community-dwelling adults aged 65 and older. In another study, participants used the Gro health app for 12 weeks, and the results showed a significant reduction in depression (*p* < .001) among participants after the intervention.

Komariah et al.'s (2022) meta-analysis assessed the efficacy of e-mental health interventions on reducing depression, showing a significant decrease between pre- and post-intervention (*p* < .001) with a pooled mean difference (MD) of 3.90, 95 % CI: 3.60–4.62), *I*^2^ = 93 %.

Malinauskas and Malinauskiene's (2022) review reported that seven reviews indicated that participants in the treatment group who received e-mental health intervention had significantly lower depression levels than the control group at post-test (SMD: −0.30; 95 % CI: −0.49 to −0.11), with a significant moderate level of heterogeneity (*I*^2^ = 71.46 %; *p* < .001; Q = 24.46).

Witarto et al.'s (2022) meta-analysis focused on the effectiveness of online mindfulness-based interventions on mental health. The findings revealed that interventions had a statistically significant small to moderate effect in reducing depression (g = −0.32; 95 % CI = −0.49 to −0.14; *I*^2^ = 0 %). Additionally, significant small effects at follow-up were observed for depression (g = −0.26; 95 % CI = −0.48 to −0.04; *I*^2^ = 0 %).

Ye et al.'s (2022) meta-analysis showed that psychosocial interventions were effective in reducing depression symptoms compared to controls (SMD = −0.80; 95 % CI = [−1.18, −0.41]; *I*^2^ = 94.75 %; 95 % CI [92.75, 96.20]; *p* < .001).

#### 3.6.3 Stress

In Bonardi et al.'s (2022) review, two authors delved into the impact of e-mental health interventions on stress reduction. One study revealed a significant reduction in stress after 2 weeks (SMD: −0.83, 95 % CI [−1.23, −0.42]), although this effect did not remain significant after 6 weeks (SMD: −0.15, 95 % CI [−0.61, 0.31]). The second study reported a significant decrease in stress after 2 weeks (SMD: 0.51, 95 % CI [0.12, 0.90]).

Hatami et al.'s (2022) systematic review summarized that one study found a notable decline in perceived stress (*p* = .43) among individuals with type I diabetes. In another study, participants used the Gro health app for 12 weeks, resulting in a significant reduction in stress (*p* < .001) among participants after the intervention.

Kurniawan et al.'s (2022) meta-analysis demonstrated that online-based interventions significantly reduced stress using the depression anxiety stress scale (DASS-21). When comparing intervention and control groups, this result shows that online-based intervention was found to significantly reduce DASS-21 scores in patients, with a pooled mean difference (MD) of −2.08 (95 % CI [−6.71, −2.55]; *p* = .001). However, the study also indicated that online-based interventions insignificantly reduced depression anxiety stress scores (a pooled MD of 0.05; 95 % CI [−2.63, 2.72]; *p* = .97) when comparing pre- and post-test scores in the intervention group.

Malinauskas and Malinauskiene's (2022) review reported that six randomized controlled trials indicated a significant reduction in stress. Post-test results showed that the treatment group with an online psychological intervention experienced significantly lower stress (*p* = .01) than those in the control group (SMD: −0.36; 95 % CI [−0.61, −0.11]). Statistically significant heterogeneity was observed (*I*^2^ = 81.99 %; *p* < .001; Q = 34.19).

Witarto et al.'s (2022) meta-analysis focused on the effectiveness of online mindfulness-based interventions on mental health. The findings revealed a statistically significant small to moderate effect in reducing stress (g = −0.62; 95 % CI = −1.09 to −0.16; *I*^2^ = 83 %). However, follow-up assessments did not yield significant effects on stress reduction.

Ye et al.'s (2022) meta-analysis showed that psychosocial interventions were ineffective in reducing stress symptoms compared to controls (SMD = −0.22; 95 % CI [−0.49, 0.04]; *I*^2^ = 78.28 %; 95 % CI [59.01, 88.50]; *p* < .001).

Other mental health outcomes were explored in these reviews. In Ye et al. (2022) systematic review and meta-analysis study, they reported that the e-mental health intervention condition was effective in reducing insomnia symptoms compared to controls a standardized mean difference (SMD) of 0.48, 95 % CI = [−0.29, −0.08]. Bonardi et al.'s (2022) systematic review found that phone call interventions were effective in reducing loneliness among participants compared to the control group (SMD = 0.48, 95 % CI [0.22, 0.74]). In D'Onofrio et al.'s (2022) systematic review and meta-analysis stated that online cognitive behavioral interventions reduced participants' intolerance of uncertainty (*p* < .01) as well as functional impairment (*p* < .01) compared with participants in the waitlist. Abraham et al.'s (2021) scoping review also touched on various mental health outcomes, including conditions related to bipolar, disruptive, impulse control, conduct, feeding, eating, obsessive-compulsive, schizophrenia spectrum, other psychotic, somatic symptom-related, and substance-related and addictive disorders.

## 4 Discussion

This study provides a thorough review of systematic reviews on the effects of e-mental health interventions in intervention types, communication channels, and mental health outcomes during the COVID-19 pandemic. By engaging in a systematic review of systematic reviews, it provides an up-to-date view of the ever-evolving landscape of e-mental health, ensuring a robust evidence base.

The predominant e-mental health intervention identified across the reviews was CBT. Several reviews consistently demonstrated the effectiveness of CBT in reducing mental health symptoms during the COVID-19 pandemic. While psychoeducation, such as online mindfulness interventions, also played a role, the reviews presented mixed findings regarding their efficacy. CBT has traditionally been the prevailing form of mental health intervention. However, in the wake of the growing popularity of e-mental health interventions, psychoeducation has gained prominence during the COVID-19 pandemic. Online psychoeducation has emerged as a compelling alternative to CBT, capturing significant attention for its potential to promote mental well-being. Psychoeducation serves as an empowering tool, equipping individuals with valuable knowledge and a deeper understanding of their mental health. For example, mindfulness-based intervention heightened self-awareness and facilitates effective coping strategies and better self-management. The online format of psychoeducation provides accessibility and convenience, allowing individuals to engage in self-help and personal growth in a way that aligns with the requirements of the digital age. This shift toward psychoeducation as an essential component of e-mental health interventions underscores its role in enhancing mental health support during unprecedented times like the COVID-19 pandemic.

The findings of this systematic review align with and expand upon the body of research on e-mental health interventions that existed prior to the COVID-19 pandemic. CBT and psychoeducation were already identified as effective tools for addressing mental health symptoms, such as anxiety and depression, through digital platforms (Donker et al., 2013; Karyotaki et al., 2018). Before the pandemic, psychoeducation was commonly employed as a supplementary tool in CBT to help individuals better understand their mental health conditions and develop coping strategies (Bauml, 2006). However, the pandemic led to a surge in online psychoeducational tools including mindfulness-based apps, webinars, and digital self-help programs designed to mitigate stress, anxiety, and uncertainty caused by the pandemic (Bonardi et al., 2022).

Communication channels used for delivering e-mental health interventions varied across the reviews. Videoconferencing emerged as the most common method, with telephone-based interventions also holding a significant presence. Additionally, mobile applications played a vital role in delivering e-mental health interventions. The varied communication channels used for e-mental health interventions highlight the need for a multifaceted approach that can cater to the diverse needs and preferences of individuals seeking mental health support. Videoconferencing and telephone-based interventions offer a human-centered experience, providing a sense of connection, while mobile applications provide self-directed options for those who value flexibility and anonymity. However, there is a pressing need for further exploration of the potential benefits associated with different communication channels for improving mental health. For example, virtual reality interventions leverage immersive virtual environments to simulate therapeutic scenarios or experiences. These interventions aid individuals in addressing post-traumatic stress disorder, practicing coping skills, and exploring mindfulness exercises. Expanding research in this direction can enhance our understanding of the efficacy and nuances of these communication modalities, while also contributing to the reduction of mental health-related stigma within the context of e-mental health interventions.

Studies conducted before the COVID-19 pandemic highlighted the availability of videoconferencing, telephone counseling, and text-based interventions (e.g., SMS reminders) as effective tools for promoting mental health (Mohr et al., 2013). While these modalities remained popular during the pandemic, their reach expanded significantly due to advancements in technology and the cancelation of traditional in person mental interventions. Prior to the pandemic, mobile applications for mental health were gaining traction but were under-explored in terms of their potential impact on mental health outcomes (Mohr et al., 2013). The COVID-19 pandemic served as a catalyst for the rapid evolution and widespread adoption of e-mental health technologies, with mobile applications becoming pivotal in promoting mental health. Additionally, immersive modalities, such as virtual reality and artificial intelligence, have begun to gain traction, though their specific effects on mental health remain understudied (Ellis et al., 2021).

The emphasis on anxiety, depression, and stress as the primary mental health outcomes in the reviews underscores the critical role of e-mental health interventions in addressing prevalent mental health challenges during the COVID-19 pandemic. Anxiety, depression, and stress are among the most common psychological struggles experienced by individuals in times of crisis and uncertainty. The consistent positive findings in these areas suggest that e-mental health interventions offer a promising solution to alleviate these symptoms and enhance the overall well-being of those affected. It is important to highlight the varying effects of e-mental health interventions, particularly in addressing stress. Witarto et al.'s (2022) meta-analysis found insignificant reduction in stress at follow-up, attributing this outcome to several factors. First, the limited number of studies included in the analysis may have constrained the findings. Second, there were notable differences in intervention duration between the two studies analyzed, one measured long-term effects over four weeks, while the other assessed a single session. Third, the researchers suggest that brief mindfulness practices may only partially alleviate stress, while longer, more sustained practices might be necessary to improve well-being. Studies by Kurniawan et al. (2022) and Ye et al. (2022) also reported insignificant reductions in stress symptoms following e-mental health interventions, although neither study offered specific explanations for these findings.

Pre-pandemic research has shown that e-mental health interventions primarily addressed anxiety, depression, and stress as the most prevalent mental health concerns (Massoudi et al., 2019). Compared with pre- pandemic research, the current study reveals a new focus to investigate the rapid expansion of e-mental health initiatives specifically designed to meet pandemic-related needs, including targeted interventions for COVID-19-specific anxiety (Bonardi et al., 2022).

## 5 Limitations

There are some limitations of the reviews that need to be addressed. First, the systematic reviews included RCTs and non-RCTs studies. Combining RCTs and non-RCTs can introduce a level of heterogeneity and variability in the data, as these study types may have different methodologies and levels of rigor. Researchers need to consider these differences when interpreting the results, as the findings from RCTs, which are generally considered more robust in terms of establishing causality than those of non-RCTs. Second, the systematic reviews included in the current study resulted in a critically poor to moderate quality based on the AMSTAR-2 criteria. It implies that significant methodological deficiencies in these reviews might lead to the issues of reliability of the findings and affect the credibility of any conclusions based on these reviews. Third, our inclusion criteria required explicit mention of COVID-19 in the articles analyzed. However, this may have inadvertently excluded relevant studies conducted during the same period that did not explicitly reference COVID-19.

## 6 Conclusions

Advancements in technology are significantly reshaping the field of mental health care, and e-mental health interventions have emerged as a crucial service, particularly during the COVID-19 pandemic. This highlights the significance of conducting a comprehensive review of systematic reviews to explore the potential effectiveness of e-mental health interventions. These findings emphasize that technology-driven approaches have the capability to effectively reduce mental health symptoms, paving the way for accessible and scalable mental health care. The review also underscores several practical implications, such as the importance of understanding and accommodating diverse communication channel modalities, including videoconferencing and telephone-based interventions, which became prevalent during the pandemic. Tailoring e-mental health interventions to specific target populations, training mental health professionals in delivering remote services, and addressing mental health outcomes are practical considerations for improving the accessibility and effectiveness of these interventions. This information is crucial for clinicians and policymakers seeking to optimize e-mental health interventions in addressing the mental health challenges that have been amplified by the COVID-19 pandemic.

## Declaration of competing interest

The authors declare that they have no known competing financial interests or personal relationships that could have appeared to influence the work reported in this paper.

## Appendix A Supplementary data

Supplementary material Image 1
